# Subacute Right Ventricular Perforation by Pacemaker Lead Causing Left-Sided Hemothorax and Epicardial Hematoma

**DOI:** 10.1155/2017/1264734

**Published:** 2017-11-16

**Authors:** Abdelrahman Ahmed, Mohamed Shokr, Randy Lieberman

**Affiliations:** ^1^Internal Medicine Department, Wayne State University/Detroit Medical Center, Detroit, MI, USA; ^2^Division of Cardiology, Wayne State University/Detroit Medical Center, Detroit, MI, USA

## Abstract

We report a case of right ventricular wall perforation by a pacemaker lead in a 78-year-old female 18 days after a permanent pacemaker insertion. This injury necessitated explant of the perforating lead and implantation of a new one with surgical backup. We review the literature and discuss the possible risk and protective factors including lead models that were associated with higher incidence of perforation. We review the traditional pacing parameters and their lack of reliability to diagnose perforation and the need for low threshold to utilize imaging in appropriate clinical scenarios. The authors believe this case is of educational value to all health care professionals, especially emergency medicine and internal medicine residents, who routinely see patients with pacemakers complaining of chest pain, shortness of breath, or dizziness.

## 1. Background

It is estimated that more than 250,000 permanent cardiac devices are implanted each year in the United States alone [1]. The incidence of asymptomatic perforations, detected by CT chest is approximately 15% for all devices, 3% for pacemakers, and 14% for implantable cardioverter defibrillators [2]. It is suggested that symptomatic perforations are even less common. Besides prolonging hospitalizations, such complications can be life-threatening [3]. Hemothorax as a subacute complication of pacemaker insertions has been seldom reported in the literature.

## 2. Case Description

A 78-year-old African American woman had a past medical history of mild dementia, left subclavian artery stenosis, hypertension, hypothyroidism, and symptomatic sinus bradycardia for which a permanent pacemaker (DDDR mode, lower rate set at 60 beats per minute) was inserted 18 days prior to her presentation. She presented to the emergency room reporting sudden onset substernal sharp chest pain, left upper abdominal pain, and mild shortness of breath. Her blood pressure was 135/97 mmHg with regular heart rate and rhythm and oxygen saturation of 95% on room air. Cardiovascular exam revealed no murmurs, gallops, jugular venous distention, or lower limb edema. Lung auscultation was significant for diminished breath sounds at left base.

## 3. Investigations

**Figure 1:**
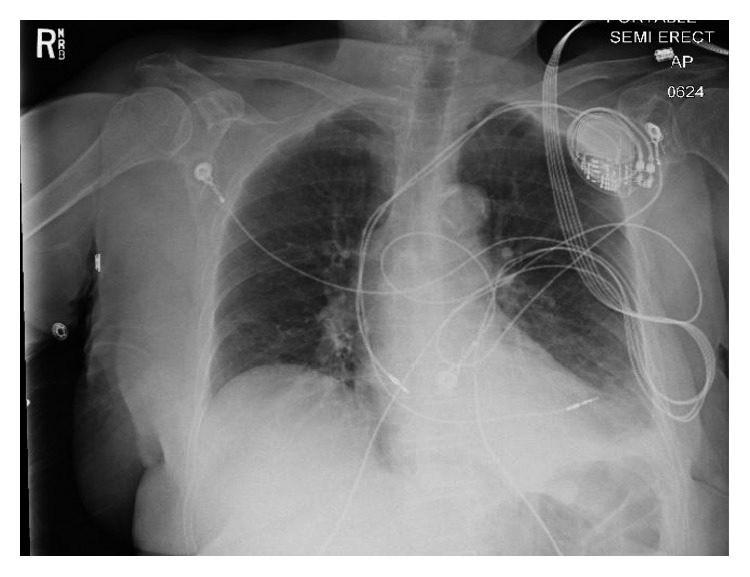
A chest X-ray of the patient on presentation revealing the right ventricular lead overlying the left.

**Figure 2:**
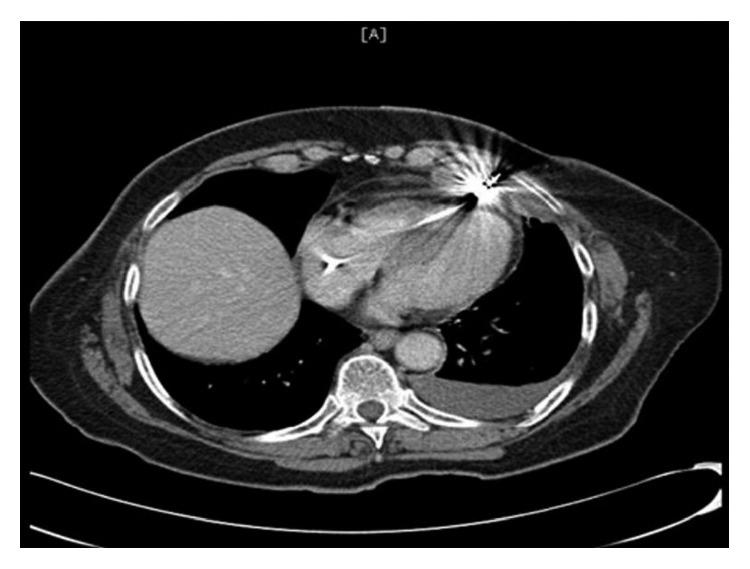
CT scan showing the tip of the right ventricular lead penetrating the anterior wall of the right.

Hemoglobin level was 13.5 gm/dl compared to 14.4 gm/dl 18 days ago. Chest X-ray showed mild left pleural effusion, and the right ventricular pacemaker lead was found to be overlying the left heart margin, raising the possibility of perforation (Figure 1). Device interrogation revealed that the lead paramaters, compared to the time of implantation 18 days ago, had changed as follows: impedance 1197 Ohms → 654 Ohms; sensing (R-wave amplitude) 11.9 mV → 18 mV; and capture threshold at a pulse width of 0.5 milliseconds remained the same at 1.0 volts. Electrocardiogram showed normal sinus rhythm, and transthoracic echocardiogram showed a hyperdynamic left ventricular systolic function with an ejection fraction of 75% and elevated filling pressure; a small heterogeneous collection in the pericardial sac was detected with no evidence of tamponade, and the lead was visualized in the right ventricle. Device interrogation revealed no change in the capture or sensing thresholds and decreased impedance (645 versus 1197 Ohms). Computed tomography (CT) of the chest revealed the tip of the right ventricular lead penetrating the anterior wall of the right ventricle and terminating in the left anterior chest wall. There was also a 2 × 5.8 cm epicardial hematoma surrounding the tip of the lead (Figure 2).

## 4. Treatment

**Figure 3:**
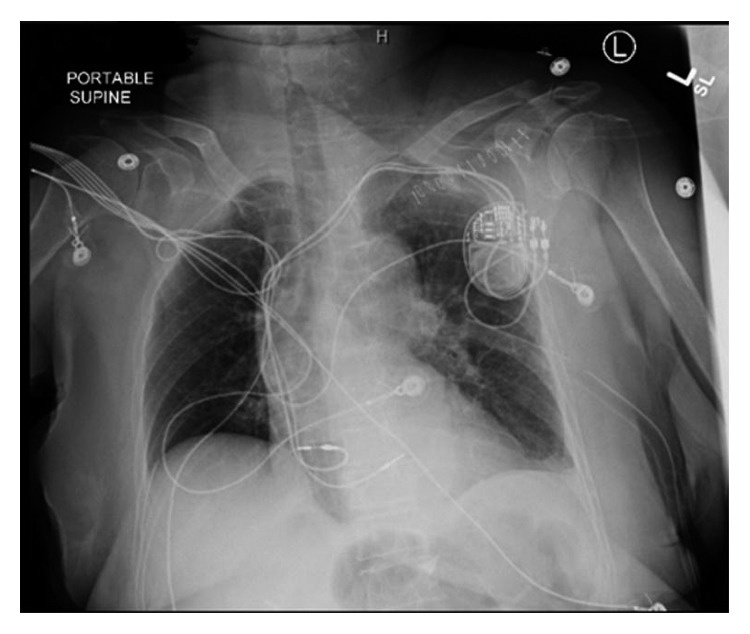
A chest X-ray showing the right ventricular lead residing at a normal position within the right ventricle after the revision.

The displaced lead was extracted, and the new lead was positioned in the midseptum (Figure 3). Excellent pacing and sensing parameters were recorded, and the lead was then fixed to the pectoralis muscle. This was done in the operating room under intracardiac echocardiogram monitoring and surgical backup. A left-sided chest tube was inserted draining over 300 ml of bloody fluid and was removed after three days.

## 5. Outcome

She had a smooth postoperative course with resolution of her symptoms and was discharged home in a stable condition and was doing well on follow-up one month later.

## 6. Discussion

Device-related ventricular wall perforations are acute, subacute, or chronic/delayed when they occur within 24 hours, one month, or more than one month after implantation, respectively [4]. Our patient suffered a symptomatic subacute perforation by the right ventricular lead following pacemaker insertion. Only few prior cases reported the occurrence of left hemothorax in cases of subacute perforation.

In a large retrospective study of 4280 patients who underwent pacemaker insertions between 1995 and 2003, Mahapatra et al. concluded that oral steroid use during the seven days preceding the procedure was the strongest predictor for cardiac perforation (HR 4.1, 95% CI 1.1–10.0, *P*=0.003), which is possibly explained by an induced myocardial atrophy [5]. Also, the use of a temporary pacemaker prior to the permanent pacemaker insertion was a significant risk factor (hazard ratio (HR) 3.2, 95% confidence interval (CI) 1.6–6.2, *P*=0.001) [3]. Other risk factors included older age and female gender, both of which are present in our case.

According to the same study, active ventricular lead fixation with helical screws seems to play a role in many cases (HR 2.8, 95% CI 1.6–4.2, *P*=0.02) [3]. However, Sterliński et al.'s study of 2247 lead implantations between January 1, 2007, and March 31, 2008, found no correlation between the perforation rate and any particular model of the implanted lead [6]. Nevertheless, Acha et al. in 2015 looked at the incidence of perforation in 72 cases implanted with Medtronic CapSureFix 5086 MRI SureScan leads, the original MRI-compatible leads, and compared them to 420 cases implanted with Medtronic SureScan leads 4076 and 5076, which were not specifically designed for MRI compatibility. Perforations occurred in 5.5% versus 0.47% of the cases, respectively (*P* = 0.005) [7]. This was partly attributed to a change in cable design and active fixation helix which was thought to increase complication rates. In another study, the perforation incidence with St. Jude Riata ICD leads was 2.6% compared to 0% with the Medtronic Sprint Fidelis leads in the same time period (*P* < 0.005) [8].

Atrial perforations are more common than ventricular perforations due to their thinner walls, and the apex is the most common site of perforation within the right ventricle. Subsequently, the use of the right ventricular outflow tract and septum as alternative sites has been suggested in high-risk patients [2, 9].

Several studies evaluated the performance of leadless pacemakers showing favorable outcomes compared to traditional pacemakers. Medtronic Micra transcatheter pacing system (TPS) and St. Jude Nanostim leadless cardiac pacemaker (LCP) reduce the risk of transvenous leads and generator pocket complications. The Micra TPS investigational device exemption study reported a 1.6% risk of perforation [10, 11]. Interestingly, the LEADLESS II study reported a similar perforation risk of 1.6% studying 527 implantations of Nanostim LCPs.

Clinical manifestations of cardiac perforations depend on the location of the perforating lead tip and vary from being asymptomatic to life-threatening pericardial effusions. The most commonly reported symptoms are chest pain, dyspnea, dizziness, and syncope [4]. Hiccups, secondary to phrenic nerve stimulation, and left chest muscle twitching, due to stimulation of the left pectoralis major by the lead tip, have been reported as well [12, 13].

In terms of diagnosis, CT scan of the chest remains a helpful adjunct to radiography and echocardiography for visualizing the lead tip and confirming the diagnosis with its ability to localize the exact site of the perforating lead tip despite the star artifact, which is a well-known artifact related to the imaging of metal implants [1]. While chest X-ray is usually the initial test performed, it may not be able to detect minimal lead migration. Nevertheless, it is still a valuable diagnostic tool being able to detect life-threatening complications associated with cardiac perforation such as pneumothorax, pericardial effusion, and large-sized hemothoraces. In addition, it is necessary to compare the lead tip position and curvature with posteroanterior and lateral chest X-rays right after the procedure [4]. Echocardiogram is not reliable given the possibility of missing the perforation altogether which was evident in our case as well.

Diagnosing a lead perforation, by utilizing the classic pacing parameters (sensing, capture, and impedence), can be misleading. Fundamentally, a lead perforation is a subset of lead dislodgement, and as such, they can share similar characteristic parameter changes. To understand the potential changes one may encounter, a brief overview is necessary.

The change in device pacing parameters depends on the location of the displaced tip. However, absence of abnormal values does not rule out perforation [2, 4]. The three parameters used are lead impedance, sensing (R-wave amplitude), and capture (pacing threshold). Lead impedance is the sum of all factors that resist the flow of electric current through the lead. The direction of change in impedance in lead perforation varies depending on where the perforating lead lies. If the lead migrates to an air-filled space, like the lung, the impedance would increase as air has more impedance than blood. If the lead ends up in a fluid-filled space, the change in impedance may not be significant [5, 14]. Sensing, measured in millivolts, is the myocardial electric signal (R-wave in the case of right ventricular leads) detected by the lead. It is important to note that the lead does not have to be in direct contact with a heart surface to detect the signals, just as EKG leads are placed on the body surface and can still detect electrical activity in the heart. Sensing values are usually expected to decrease, and this case, to the best of our knowledge, is the first in the literature where sensing values have increased. It is worth noting that this value can significantly be affected by the alignment of the lead with the electric current vector. The more parallel the two, the larger the R-wave is, and thus if the lead's new position after migration was more parallel to the electric current vector, this R-wave can be larger in value. Pacing threshold (capture) is the minimal amount of energy required to detect electrical activity in the myocardium. The smaller this value, the better as IT ( add IT) saves the battery life of the system. In case of lead perforation and migration, the change in capture threshold depends on the distance between the migrated lead tip and the initial area of implantation. A lead piercing the heart may not travel a long distance, leading to almost unchanged pacing threshold, and a lead migrating through the pulmonary artery traveling a longer distance can have capture failure.

Changes in pacing parameters suggest lead dislodgement. A perforation represents a specific type of dislodgement with mortal potential. At the time of implantation of pacing leads, the implanter may elect to manually dislodge his lead due to an unacceptable parameter (sensing, capture, or impedance) and move to a more acceptable position. During implantation, this was elective; similarly, a lead can spontaneously dislodge to a position that has similar, worse, or even better parameters than the original implant site. No specific parameter will necessary be inclusive or exclusive of the specific type or position of the dislodgement, nor can it differentiate between lead dislodgement and perforation. Once lead perforation or dislodgement is suspected, it is imperative that the patient undergoes imaging to differentiate between the two.

To conclude the discussion about pacing parameters, the following rules should always be considered:

1. Change in pacing parameters suggests lead dislodgement (not necessarily lead perforation).
2. Lack of change in pacing parameters does not exclude lead dislodgement (or perforation).
3. There is no consistent lead pacing parameters to rule in or out dislodgement/perforation.
4. Once a change in parameter is detected and lead dislodgement is suspected, clinicians should proceed to imaging studies to differentiate between lead dislodgement and perforation.

Table 1 summarizes the expected changes in pacing parameters when pacemaker leads perforate the heart wall and also explains why these changes are inconsistent.

Management strategies include lead repositioning, lead extraction, or open heart surgery. In hemodynamically stable patients, the preferred strategy is lead extraction under close echocardiographic monitoring with surgical backup followed by new lead placement in a different location. Interestingly, prior case reports described the successful use of fibrin glue patch or cyanoacrylate glue, injected through the pericardial space, to seal RV perforation secondary to pacemaker leads [15, 16]. In cases of hemodynamic instability, rapidly progressive pericardial effusion, or injury of surrounding organs, surgical management is the recommended treatment [1]. Some studies suggest that the extraction of a chronically perforated lead, without neither device malfunction nor resulting symptoms, is not mandatory [2].

## Figures and Tables

**Table 1:** 

	Normal values	Expected change in perforation	Why the parameter may change differently even in perforation
Impedance	400–1000 Ohms	Usually decreased	May increase if the lead ends in an air-filled space
Sensing (R-wave amplitude)	At least 5 mV	Usually decreased	May increase if the lead becomes parallel to the incoming electric current vector
Capture threshold	Less than 1 volt at a pulse width of 0.5 milliseconds	Usually there is loss of capture	May remain the same if the lead has not moved a long distance from the heart
